# On-demand Doppler-offset beamforming with intelligent spatiotemporal metasurfaces

**DOI:** 10.1515/nanoph-2023-0569

**Published:** 2023-11-17

**Authors:** Xiaoyue Zhu, Chao Qian, Jie Zhang, Yuetian Jia, Yaxiong Xu, Mingmin Zhao, Minjian Zhao, Fengzhong Qu, Hongsheng Chen

**Affiliations:** ZJU-UIUC Institute, Interdisciplinary Center for Quantum Information, State Key Laboratory of Extreme Photonics and Instrumentation, Zhejiang University, Hangzhou 310027, China; ZJU-Hangzhou Global Science and Technology Innovation Center, Key Laboratory of Advanced Micro/Nano Electronic Devices & Smart Systems of Zhejiang, Zhejiang University, Hangzhou 310027, China; Jinhua Institute of Zhejiang University, Zhejiang University, Jinhua 321099, China; Department of Information Science and Electronic Engineering, Zhejiang University, Hangzhou 310027, China; Ocean College Zhejiang University, Zhoushan 316021, China

**Keywords:** spatiotemporal metasurfaces, Doppler effects, inverse design and deep learning, wireless communications

## Abstract

Recently, significant efforts have been devoted to guaranteeing high-quality communication services in fast-moving scenes, such as high-speed trains. The challenges lie in the Doppler effect that shifts the frequency of the transmitted signal. To this end, the recent emergence of spatiotemporal metasurfaces offers a promising solution, which can manipulate electromagnetic waves in time and space domain while being lightweight and cost-effective. Here we introduce deep learning-assisted spatiotemporal metasurfaces to automatically and adaptively neutralize Doppler effect in fast-moving situations. A tandem neural network is used to establish a rapid connection between on-site targets and time-varying series of spatiotemporal metasurfaces, endowing the capability of on-demand beamforming with Doppler effects offset. Moreover, oblique incidence problems are also studied in practice, which can be used for relieving multipath effect. In the microwave experiment, we fabricate the intelligent spatiotemporal metasurfaces and demonstrate the potential to fulfill Doppler-offset beamforming under oblique incidence.

## 1 Introduction

When travelling in a high-speed train, we often encounter a vexing phenomenon that someone yells repeatedly on the phone, “I am losing you, say it again.” The uncomfortable noise reverberates in the compartment, as one falls. Such a phenomenon often takes place in the scenarios where the relative velocity between the user equipment and base station is very high. The physical essence is that the generated Doppler effect shifts the frequency of the transmitted signal, making it unmatched with the received electronic devices [1]. To solve this, evaluating and mitigating the Doppler frequency shift becomes crucial. In recent decades, significant efforts have been devoted to designing advanced communication systems that can operate in extreme conditions and enhance the overall performance of the proposed standards [1, 2]. Traditional techniques mainly rely on signal handover and processing methods, typically employing moving relay nodes (MRNs) attached to train rooftops to facilitate handover [2–4]. However, such techniques are complex, costly, and have terrible performance. Advanced digital signal, channel processing, and modulation methods are also utilized to mitigate Doppler effects [3–7]. Nonetheless, these techniques are limited to low velocities and may not be applicable to complex scenarios, where Doppler effect may be compounded with other challenges, such as multipath effects and everchanging circumstances.

Recently, metasurfaces have emerged as a promising technology for arbitrarily manipulating the amplitude, phase, and polarization of electromagnetic (EM) waves in multiple dimensions [8, 9]. They feature significant advantages, such as low cost, lightweight, and easy fabrication, making them attractive for a wide range of applications [10], including meta-lenses [11], invisibility cloaks [12], terahertz [13, 14], polarization modulators [15–18], gain devices [19], wireless communications [20, 21], and other functional applications [22–26]. As a new member of metasurface family, spatiotemporal metasurfaces are even more versatile [27–29]. Based on reconfigurable metasurfaces [30–34], spatiotemporal metasurfaces are capable of generating a constellation of harmonic waves, extending EM manipulation from the spatial-only domain to the space-time domain and facilitating numerous applications [35–38]. By deliberately designing the time-varying series, harmonics can be used to mitigate Doppler effect in fast-moving communication scenarios [39–41]. In doing so, finding the desired spatiotemporal matrices composed of time-varying series typically involves exhaustive or heuristic algorithms, which are time- and resource-consuming and prone to falling into local optima. However, in on-site Doppler-offset beamforming scenes, situational information tends to be nonstationary [1, 2, 6, 41], which means search-and-save methods do not fit these high-mobility scenarios. Besides, conventional algorithms inevitably discard many unmatched results, causing a great waste of computing resources. Moreover, existing works are mainly based on vertical incidence. While, oblique incidence is inevitable in practice and can be leveraged to relaxing multipath effects. Given these factors, developing an effective approach to avoid time-consuming searches, endow spatiotemporal metasurfaces intelligence, and ease oblique incidence is of great significance in fast-moving situations. Recent advances in inverse design [42–47], deep learning [48–52] and give out some hints.

In this work, we present and experimentally demonstrate an intelligent spatiotemporal metasurface for on-demand Doppler-offset beamforming. The design involves manipulating reflected waves to compensate for the Doppler effect, which dynamically changes as users are moving. Additionally, reflected beams need to be manipulated adaptively for signal transmission. To achieve this, deep-learning assisted inverse design is constructed. To mitigate the non-uniqueness issues [44, 47, 49, 53], a tandem deep neural network [53–55] is adopted. The equivalent states of spatiotemporal metasurfaces are also analyzed to facilitate network training. For practical generality, we discuss and validate the adaptively arbitrary beamforming ability of the system. Furthermore, oblique incidence situations are also analyzed for possible outdoor applications and potential relief of multipath effects that inevitably accompany Doppler effects and causes graver fading. All experimental results prove that the proposed metasurfaces can adaptively compensate for signal loss caused by the Doppler effect in dynamic situations. This work represents a significant breakthrough in wireless communication and highlights the potential of intelligent spatiotemporal metasurfaces in various applications [12, 16, 29, 38, 40, 41, 47, 56], [57], [58].

## 2 Results

### 2.1 Concepts

**Figure 1:**
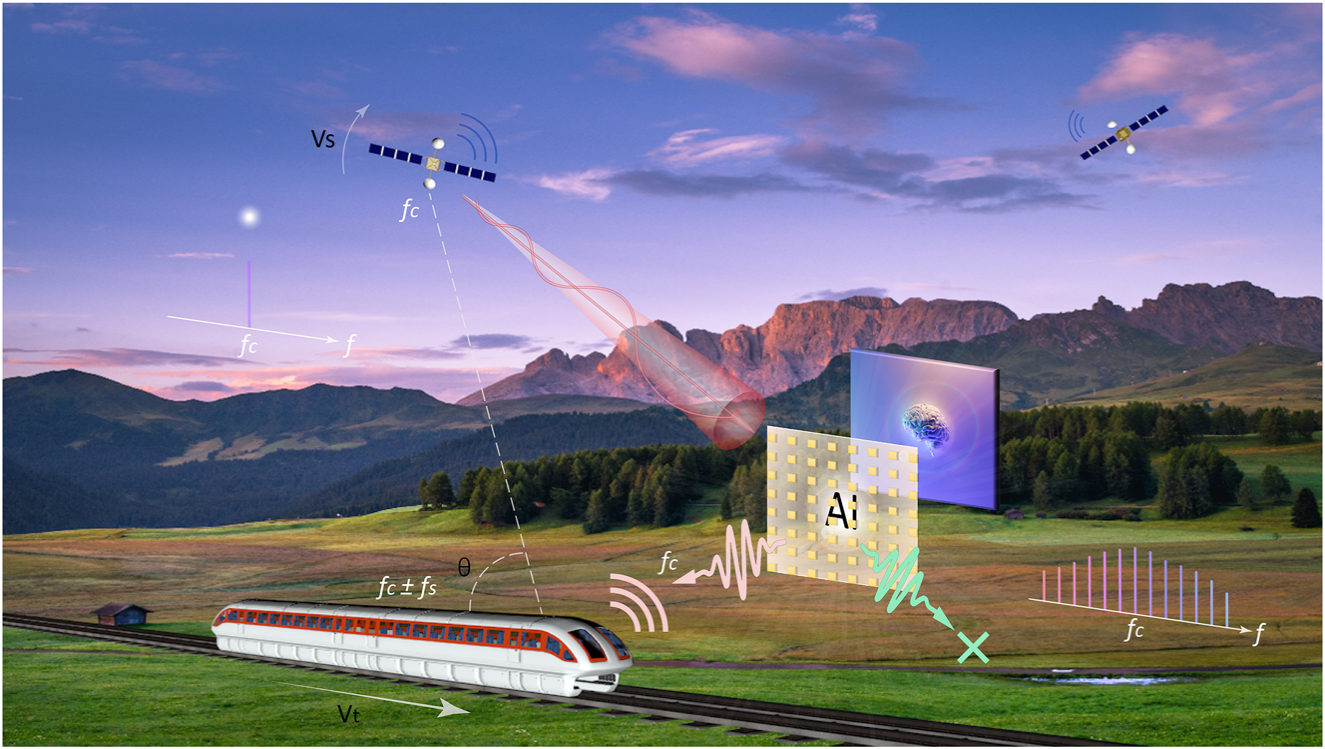
A conceptual illustration of intelligent spatiotemporal metasurfaces for on-site elimination of Doppler effect in fast-moving scenes. Assuming that the satellite transmitting a carrier signal to the train at a frequency of fc and moving at a speed of Vs, with the train moves at a speed of Vt, the signal will suffer from a frequency shift fs. This frequency shift can be compensated via intelligent spatiotemporal metasurfaces deflections. The metasurfaces can introduce a plethora of harmonics into the signal for Doppler compensation. With the help of deep learning and inverse design methods, beamforms of the harmonics can be adaptively manipulated to users in trains (pink path). And by introduce oblique incidence analysis and offsets, signals from the other satellite will be decreased and reflected to other directions (green paths).

Doppler effect is a phenomenon that occurs when there is a relative motion between a source of waves and an observer, causing a frequency change at the received end [1, 2, 39]. In fast-moving communication situations, such as those involving moving vehicles or aircraft, the Doppler effect greatly degrades the accuracy and reliability of communication signals, especially when multiple subcarrier techniques are involved [6]. As illustrated in Figure 1, the frequency shift *f*_*s*_ is related to the relative velocity *v*_*t*_ and angle *θ* between the transmitter and receiver [39, 40], and can be calculated via:

(1) $$f_{s}=\frac{V_{t}}{c}*f_{c}*\cos\theta$$

Herein, *f*_*c*_ represents transmitted carrier frequency (incident wave frequency), and *c* denotes speed of light. Supposing there is a satellite transmitting messages to a train at a carrier frequency of 3 GHz and moving at a speed of about 7 km/s, the maximum frequency shift will be 70 kHz. As for high-speed trains usually moving at speeds about 360 km/h, the maximum frequency shift will attain 1 kHz [1, 6]. Since the angle *θ* is varying, the frequency shift *f*_*s*_ keeps changing.

To mitigate the Doppler effect, we use intelligent spatiotemporal metasurfaces as intermediate media. Once illuminated by incoming carrier wave, spatiotemporal metasurface generates a constellation of controllable harmonics [27–29]. As shown in Figure 1, these harmonic waves have arithmetic frequency shifts away from incident frequency (*f*_*c*_). By adjusting the tuning speed and pulse numbers, the frequency shift can be altered as desired [38], thereby eliminating the frequency shift caused by the Doppler effect [39]. In addition, Doppler shifts in outdoors are often accompanied with multipath effects. In our approach, we introduce an oblique incidence compensation methodology to ease this affliction. This technique selectively compensates for a desired incident path, while concurrently reflected undesired paths to dead zones as illustrated in Figure 1.

In practical applications, the metasurfaces can be deployed by the roadside like a wireless relay node as illustrated in Figure 1. In this case, the transmitted signal should cover the metasurface to generate frequency-shifted waves. Sometimes the Doppler-offset signal and the original input signal (with Doppler effect) might both exist, the latter of which can be blocked by metal like train’s roof or directly filtered. Nonetheless, the Doppler-offset signal is now generated physically, thus offering a new choice to improve communication services. Moreover, in practical applications, the Doppler-offset signal can be amplified to largely surpass Doppler signal by introducing gain metasurfaces [19]. In order to ensure the reflected signal can be received, main beams of the reflected waves are automatically steered to active users with the help of deep learning and inverse designs. The metasurface may also be deployed inside the train to act as a moving processing device. Similar to other indoor metasurface deploying schemes, the metasurface can be attached to the inwalls in a train. Signals emitted from base stations impinges on the metasurfaces by passing through windows, and then is reflected to the desired direction with frequency shifts compensated.

### 2.2 Spatiotemporal metasurfaces

**Figure 2:**
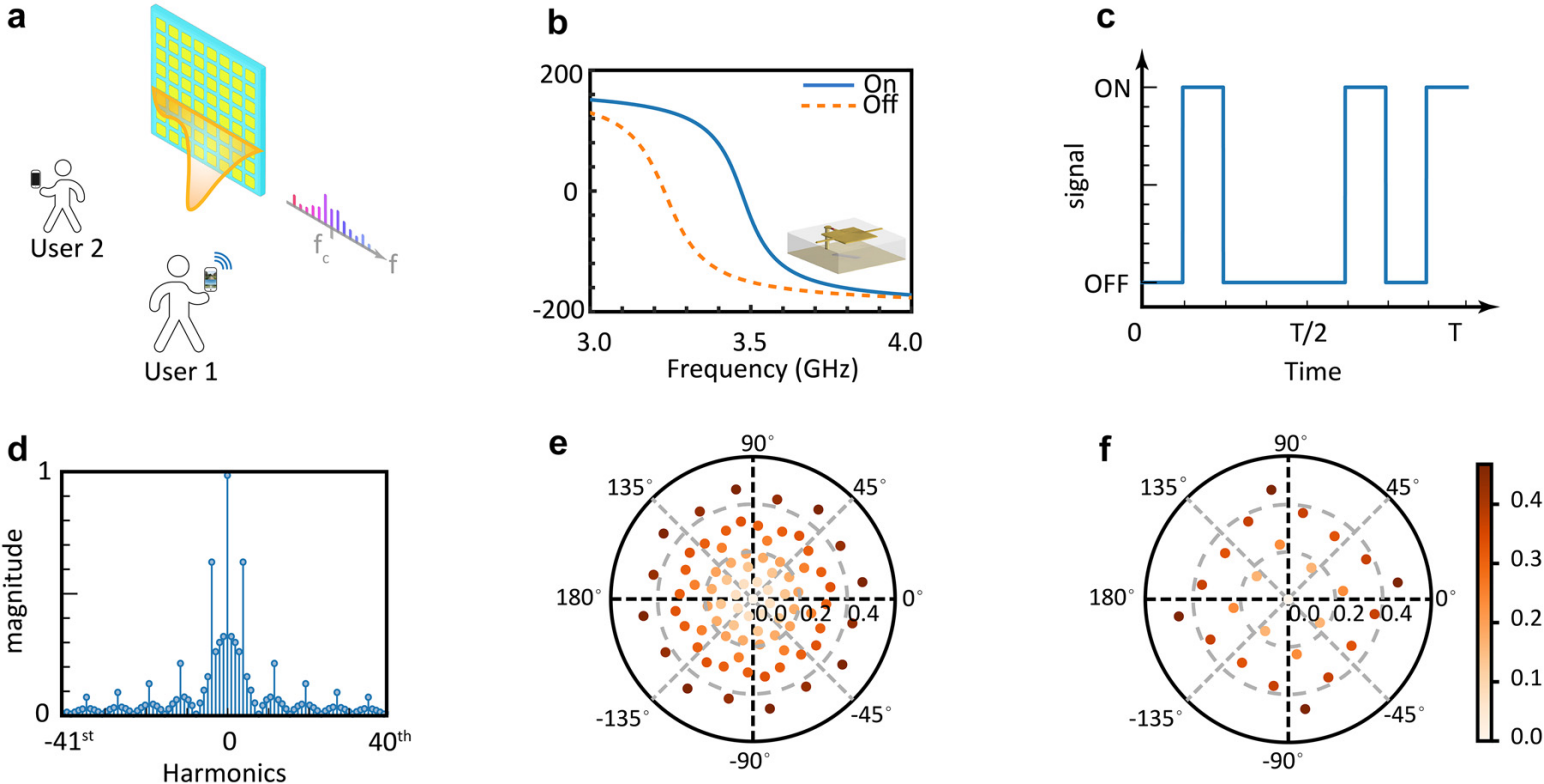
Design and equivalent states of spatiotemporal metasurfaces. (a) Spatiotemporal metasurfaces can generate com-like frequency harmonics whose beams can be manipulated to be oriented to working users, on induced by a monophonic wave. (b) Simulated frequency response of metasurface unit cell under ON/OFF state. A diagram of a cell structure illustrated at the right bottom in the figure. (c) A diagram of periodic signal for demonstration. (d) The decomposed frequencies of the periodically varying signal. (e) All possible equivalent reflection coefficients for the +1st harmonics. (f) All possible equivalent reflection coefficients for the +2nd harmonics.

As showcased in Figure 2a, spatiotemporal metasurfaces can generate controllable comb-like harmonics whose beamforms are manipulatable. The fabricated metasurfaces are composed of 64 tunable unit cells employing basic square structure [31]. More details about the metasurface and unit cells designs are left in Supplementary Note 1. By switching the voltage between high and low, the unit cell will work at ON and OFF states [31, 38]. As illustrated in Figure 2b, the two states bear obvious phase differences. Experimental measurements are also conducted to catalog the reflection amplitude and phase at the ON/OFF states; see Supplementary Note 1. Here we want to mention that although reflected amplitude at ON state is relatively small, the final results are still acceptable as proved in next sections especially in the experiment part. Such amplitude suppression extensively exists in microwave reconfigurable metasurfaces, which is in large part due to the inherent resistance of diode integrated in the metasurfaces.

When the controlling voltages vary periodically, the reflection coefficient will show synchronously periodical transitions between the ON and OFF states. Hence, derived reflection coefficient can be deemed as time-varying series, articulated as:

(2) $$f\left(t\right)=\overset{\infty }{\underset{n_{0}=0}{\sum }}\overset{n_{0}L-1}{\underset{l=n_{0}}{\sum }}\left[\Gamma_{l}G_{l}\left(t\right)\right]$$

wherein *L* denotes the total number of states in a period. And *n*_0_ is a natural number. $G_{l}\left(t\right)$ represents a rectangular pulse, which is defined as:

(3) $$G_{l}\left(t\right)=\left\{\begin{array}{cc} 1,\; & \left(l-1\right)T/L\leq t<lT/L\\ 0,\; & \text{else} \end{array}\right.$$

where *T* represents the time duration of each period. Based on Fourier theorems, a periodic signal can be decomposed into a summation of sine and cosine waves with varying frequencies [28, 35, 48]:

(4) $$f\left(t\right)=a_{0}+\overset{\infty }{\underset{n=1}{\sum }}\left[a_{n}\cos\left(n\omega t\right)+b_{n}\sin\left(n\omega t\right)\right]$$

In this equation, $f\left(t\right)$ represents the periodic function, and *ω* denotes the fundamental angular frequency, which can be calculated by *ω* = 2*π*/*T*. Therefore, the fundamental frequency shift is *f*_1_ = 1/*T*, and other harmonic frequencies are integer multiples (±1, ±2, …) of *f*_1_.

To offer a more intuitive understanding of how harmonics are generated, we choose a time-varying signal composed of a 0–1 series for demonstration as illustrated in Figure 2c. The corresponding spectrum is computed using fast Fourier transformation (FFT) methods and depicted in Figure 2d. The figure showcases that the time-varying series introduce a plethora of higher-order harmonics as previously described. As for the reflection coefficient time-varying series, we simply need to replace 0/1 in the signal with ON/OFF reflection coefficients respectively.

The Fourier decomposition of the series $f\left(t\right)$ can also be expressed in a complex exponential form as follows:

(5) $$f\left(t\right)=\overset{\infty }{\underset{n=-\infty }{\sum }}\left[c_{n}\exp\left(jn\omega t\right)\right]$$

where *c*_*n*_ signifies the complex coefficient for each component, encompassing the amplitude and phase of the corresponding harmonic wave. In essence, *c*_*n*_ acts as the equivalent coefficient of each harmonic wave and is determined by the reflection state configurations within a specific time period. Consequently, for spatiotemporal metasurfaces, numerous equivalent reflection coefficients exist for each harmonic wave, with the precise value contingent on the time-varying series. Notably, these equivalent reflection coefficients function as real reflection coefficients.

To illustrate this concept, we depict all possible equivalent reflection coefficients for the +1st and +2nd harmonics (*L* = 8) in provide Figure 2e and f. Evidently, a plethora of selectable equivalent states exist for each harmonic. To minimize the loss effect, it is advisable to utilize the equivalent states with the highest equivalent magnitudes. However, the same equivalent state may be induced by different time-varying series. This one-to-many problem leads to an ill-posed deep neural network that is incapable of converging, known as non-uniqueness [44, 47, 49, 53].

### 2.3 Neural network and inverse design

**Figure 3:**
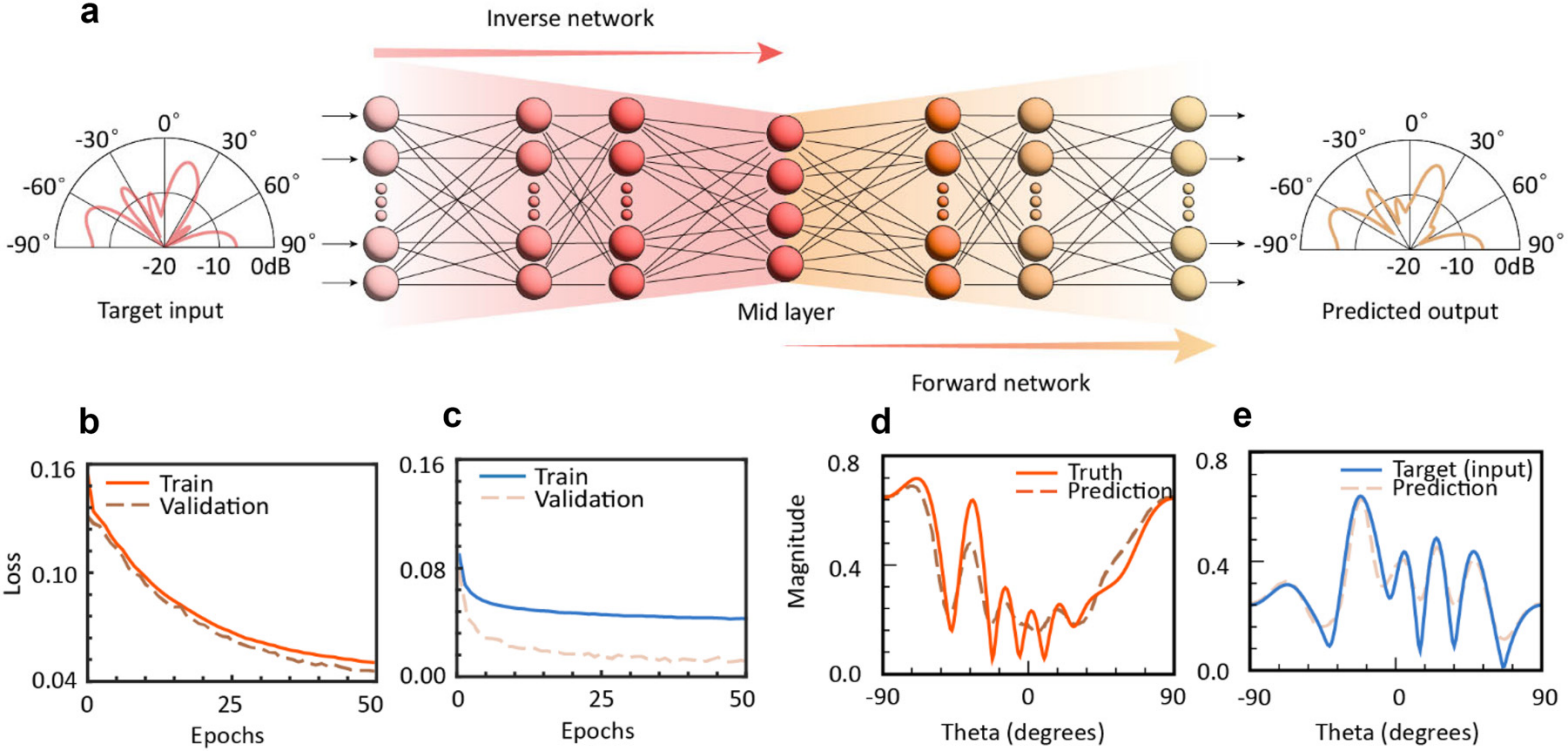
Network training and evaluation. (a) Network structure. The whole network is composed of the forward network and inverse network. The input and output network are both far-field, and the desired equivalent state arrangements can be extruded from mid layer. (b) The training loss result of the forward network. (c) The training loss of the whole tandem network. (d) Test results of the forward network. (e) Test results of the tandem network.

In our approach, we leverage deep learning and inverse design techniques [44], [45], [46], [47, 51], where the desired target far-fields are used as inputs while the associated reflection state arrangements act as outputs. However, same field can be produced by different equivalent state arrangements, which give rise to non-uniqueness issue. To tackle this challenge, tandem deep neural network architecture [53–55] is adopted as depicted in Figure 3a. This architecture is crafted to effectively yield optimal equivalent spatiotemporal state arrangements fulfilling the desired target fields patterns. The forward network is composed of six hidden layers, which have 128, 256, 512, 512, 256, and 128 nodes, respectively. While, the inverse network is made up of five hidden layers, containing 128, 256, 512, 512, and 512 nodes. The training process begins by pre-training forward network to predict far-fields of a given state arrangement. Subsequently, this pre-trained forward network is integrated into the inverse network for joint training. Upon the completion of training, the inputs and outputs of entire network should be similar, with equivalent spatiotemporal state arrangements being able to be extruded from mid-layer. As the equivalent states are closely associated with time-varying series, the inverse network has the capability to autonomously devise time-varying series arrangements to meet specified target fields. The training losses of forward and tandem networks are illustrated in Figure 3b and c, respectively. A consistent decline in the loss values across epochs is evident, signaling the convergence of the networks. Terminal losses for the forward and inverse networks are approximately 0.06 and 0.08, respectively, which indicates a successful training process.

To ascertain the efficacy of the training, we implemented stringent evaluations of both the forward and tandem networks. Test datasets were comprised of randomly generated arrangements and their respective ground-truth far-fields. The ground-truth far-fields were computed numerically, employing well-established calculation methods grounded in antenna theories and related studies [12, 21, 42]. For intuitive understanding, predicted fields, obtained from forward network’s output layer (Target outputs), were plotted in Figure 3d with corresponding ground-truth fields for contrast. Results showcase that the forward network can predict the accurate field response of given arrangements. Figure 3e is about the comparation of fields predicted by tandem network and target fields, implying that the tandem network can generate feasible arrangements of desired fields. This juxtaposition demonstrates that the methodology could generate equivalent state arrangements in accordance with specific target far-field demands.

### 2.4 Arbitrary beamforming and oblique incidence compensation

**Figure 4:**
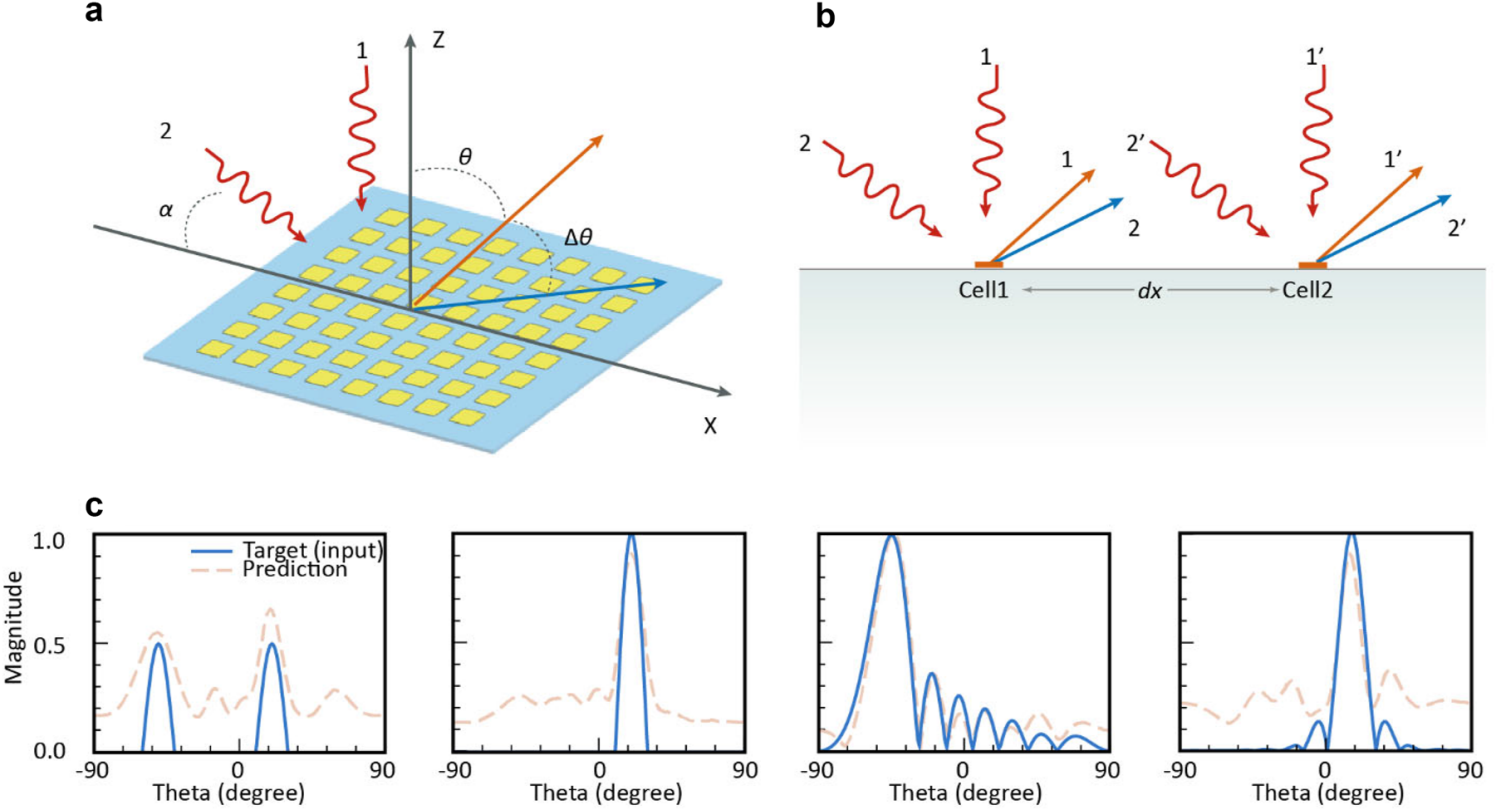
Oblique incidence compensation analysis diagrams and arbitrary beamforming demos. (a) The 3D view diagram of reflection situation analysis with oblique incidence. (b) A planar view between two adjacent cells under oblique incidence. (c) Some demos for arbitrary beamforming capability and methods.

In practical applications, conditions often deviate from the ideal, and the objectives might be less specific such as only directional information is known [21, 26, 36], or multiple beams or sidelobes are in demands. Here we can feed the network with on-demand target fields which are fabricated via adjusting truncated sin functions or rolling-cosine functions. As showcased in Figure 4c, predicted fields can generally depict the tendency of target field trends and fit the targets at main beams/peaks. This flexibility is critical in ensuring that the system is attuned to the dynamic nature of practical environments and is capable of catering to a spectrum of requirements.

The angle of incidence can also have a significant impact on the performance of the system. In practical environments, incident waves are often oblique [1–6], whereas most field calculations are fundamentally predicated on vertical incidence scenarios [26, 38]. This discrepancy can lead to the harmonic beamforms deviating from the desired direction, which would have been calculated assuming vertical incidence. Notably, while it is feasible to manipulate the metasurfaces to reflect an incoming plane wave in a specific direction when it impinges vertically (as in wave 1), the situation becomes complex when the incoming wave is oblique (as in wave 2). In this latter scenario, if no adjustments are made to the metasurfaces, the wave must be reflected in a different direction, in accordance with the generalized Snell’s law [8].

To address this challenge, we can consider the impact of oblique incidence on the system. Specifically, the oblique incoming wave imparts an additional phase difference between adjacent unit cells, as depicted in Figure 4b. When the spatial distance between two neighboring unit cells is denoted as d*x*, the incoming wave impinging on the right unit cell will lag in phase compared to the left unit cell, resulting in an additional spatial phase difference. Referring to antenna theory, this additional phase difference can be computed via the subsequent equation:

(6) dΦ=2πk⋅dx⋅cos⁡α

where dΦ represents the additional phase difference, *k* denotes the wave number, d*x* is the spatial distance between two adjacent unit cells, and *α* signifies the incident angle of the incoming wave. Given that this additional phase difference can be precisely calculated, it is possible to adjust the predicted time-varying series arrangements by incorporating additional equivalent state phases to compensate for these additional phase differences. Consequently, by compensating for this additional phase difference, the metasurface design can be refined to counteract the impacts of oblique incidence, ensuring that the harmonic beams retain their desired directions irrespective of the angle of incidence. This adaptability bolsters the system’s versatility and robustness, rendering it more adept for a diverse array of practical communication scenarios. Moreover, as above-mentioned, the arbitrary beamforming combined with oblique incidence compensation can be utilized to ease multipath effects, as undesired paths owning different incident angels and can be deflected to dead zones with main beams of desired paths oriented to working users.

## 3 Experiments

**Figure 5:**
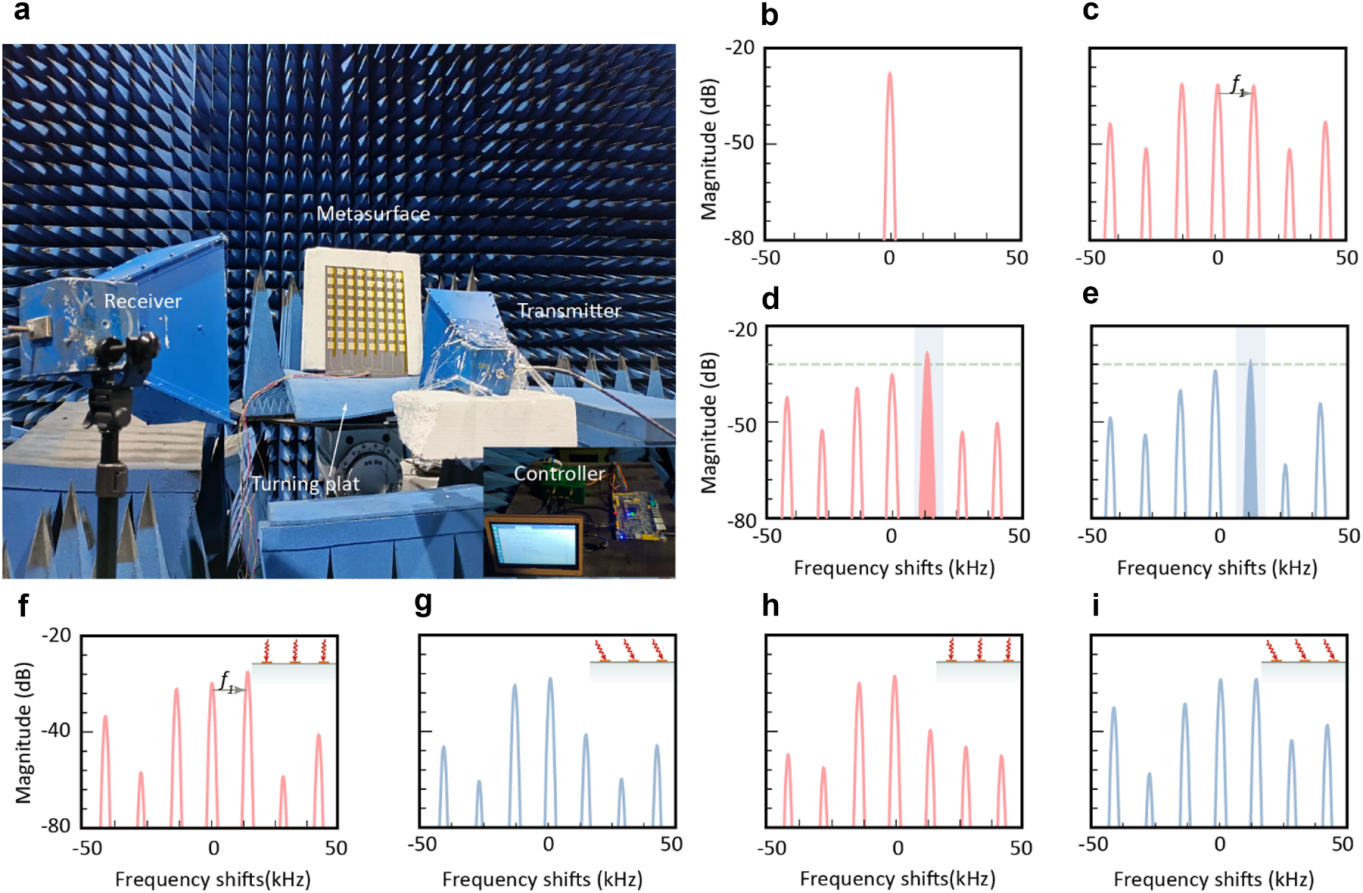
Experimental results. (a) A photograph of used experimental setup. (a) Received spectrum under vertical incidence with no modulation. (b) Received spectrums under vertical incidence with metasurface time modulated. The frequency *f*_1_ is the frequency difference used for Doppler effect compensation. (c) Received spectrum under vertical incidence with no modulation. (d) Received spectrums under vertical incidence with target beam steered to *θ* = +45° where the receiver is placed. (e) Received spectrums under vertical incidence with target beam steered to *θ* = −45° where the receiver is placed. (f) Received spectrum under a vertical incident scene. (g) Received spectrum under oblique incidence, with other conditions and setups being same as that of Figure 5f. And the incident angle *α* is 60°. (h) Received spectrum under a vertical incident scene, with time-varying series altered to compensate for oblique incidence. (g) Received spectrum under oblique incident scene, with time-varying series altered to compensate for oblique incidence situations where the incident angle *α* is 60°.

To ascertain the viability of the proposed methodology, experiments were executed in a standard microwave anechoic chamber [12, 28, 42], as illustrated in Figure 5a. The experimental arrangement incorporated two broadband, linearly polarized, double-ridged horn antennas; one functions as transmitter while the other acts as receiver. The transmitter was interfaced with microwave signal generator, emitting plane waves at a singular frequency. Concurrently, the receiver, connected to a spectrum analyzer, was employed to scrutinize the spectral composition of the signals reflected, with an intelligent controller providing controlling time-varying series (voltages). In scenarios involving vertical incidence, the metasurface was strategically positioned at the center, facilitating the analysis of reflected signals at diverse angles. For oblique incidence scenarios, the transmitter was repositioned to align with the specified directions. More descriptions upon the systems can be found in Supplementary Notes 3.

As previously analyzed, spatiotemporal metasurfaces possess the capability to generate controllable harmonics which is instrumental for Doppler cancellations. To verify this, we initially impinge a monophonic plane wave vertically onto the metasurfaces. Assuming that a frequency shift of 15 kHz is required for Doppler cancellation and the desired compensated frequency is 3.5 GHz + 15 kHz, we employ the transmitter emitting a 3.5 GHz plane wave to simulate the raw signal, with the metasurface unmodulated. Subsequently, the receiver captures unprocessed signal, as illustrated in Figure 5b. When the metasurfaces modulated, the reflected wave exhibits a comb-like spectrum, as depicted in Figure 5c. Notably, there is a frequency shift of *f*_1_ (15 kHz) between adjacent harmonics, which can be harnessed for Doppler cancellation.

Following the initial tests, we proceed to conduct another experiment for ascertaining the metasurfaces capability of manipulating beam directionality. In this experiment, target beamforms were configured to peak at the directions of *θ* = ±45°. The target details are explained in Supplementary Notes 2. For the sake of consistency, we opted to use the +1st harmonic as a representative example for demonstration purposes. The receiver was strategically placed at these two directional positions to capture the reflected harmonics. The spectrum of the signals received was then plotted in Figure 5d and e. The results reveal that the +1st harmonic is significantly elevated compared to the other harmonics in the received spectra, indicating that the energy can be effectively transmitted in this specific direction with the frequency shift being efficiently compensated. As aforementioned the little decrease of cell reflection magnitude dose not severely influent final results compared with no harmonics generated.

Additionally, the results can be employed to counteract the deviations introduced by oblique incidence. To experimentally validate this, the metasurface was subjected to vertical and oblique (*α* = +60°) impingement for contrast, keeping the receiver stationary at the same position. Here, the target was fed into the network to render the +1st reflected harmonic peaking at the direction of *θ* = +45° under vertical incidence. Spectrum of the received signal is depicted in Figure 5f. Subsequently, the incident angle was modified to *α* = +60° as stated, while the receiver remained relative stationary to the metasurface. The spectrum of the received signal under these conditions is illustrated in Figure 5g. It is noteworthy that the distances between the metasurface and both the transmitter and receiver remained stable in this experiment. As clearly evidenced in Figure 5f and g, that incident angles that do not match the desired configuration will results in a reduction in the magnitude at working harmonics. This observation is in consonance with the preceding analysis that different paths carry different additional phase differences that lead to a directionally selective fading.

When the additional phase difference is compensated, the selected paths will be changed. To be specific, obliquely incident paths can now transmit signals like the previous vertically incident one, with the vertically incident path undergoing magnitude fading at the compensation frequency. For verification, the efficacy of the proposed compensation technique for oblique incidence is subsequently evaluated through experiments. As depicted in Figure 5h and i, the magnitude of the received +1st harmonic under vertical incidence is now diminished, while that under oblique incidence is augmented. Consequently, the path with oblique incidence becomes conducive to signal propagation, while the vertically incident path is attenuated. In essence, this approach establishes the capacity for path selection, which can be utilized to ease multipath effects. Here we want to mention that aperture of the metasurface should be carefully designed to improve signal gains to enhance the reflected signal intensity. Generally speaking, the gain increases with effective aperture enlarging referring to antenna theories as depicted by the equation: *G* = *πD* ^2^ *η*/4, where *G* is the aperture gain, *D* ^2^ is the area of the aperture and *η* is aperture efficiency. In outdoors, the size can be enlarged to offer more signal gains by using more unit cells, as long as the wave impinged on the metasurfaces is still far-field.

## 4 Conclusions

In conclusion, we introduce smart spatiotemporal metasurfaces designed to eliminate the Doppler effect and multipath effect in fast-moving environments. The approach incorporates tandem neural network to control it. Deep learning and inverse design enable the spatiotemporal metasurfaces to adapt rapidly and automatically to various situations. On-demand beamforming endows the generality and feasibility in ever-changing scenes. Oblique incidence compensation methods offer a new way to relieve multipath effect by leveraging the incident phase difference of different paths, making the whole system more applicable in outdoor scenes. Experiments conducted under both vertical and oblique incidence showcase the effectiveness of the proposed scheme. This work paves the way for future research and applications of spatiotemporal metasurfaces in fast-moving scenario communications, meriting a series of relative researches and applications such as cloaking [12, 13, 42], smart cities [21, 26], and intelligent metamaterials [47, 49, 51, 56, 58].

## Supplementary Material

Supplementary Material Details

## References

[1] Mach P., Becvar Z., Plachy J. (2022). Mitigation of Doppler effect in high-speed trains through relaying. *2022 IEEE 95th Vehicular Technology Conference: (VTC2022-Spring)*.

[2] Ma R., Cao J., Feng D., Li H., He S. (2020). FTGPHA: fixed-trajectory group pre-handover authentication mechanism for mobile relays in 5G high-speed rail networks. *IEEE Trans. Veh. Technol.*.

[3] Zhang J., Du H., Zhang P., Cheng J., Yang L. (2020). Performance analysis of 5G mobile relay systems for high-speed trains. *IEEE J. Sel. Area. Commun.*.

[4] Chen Y., Niu K., Wang Z. (2021). Adaptive handover algorithm for LTE-R system in high-speed railway scenario. *IEEE Access*.

[5] Yu X. C., Luo Y., Chen X. J. (2016). An optimized seamless dual-link handover scheme for high-speed rail. *IEEE Trans. Veh. Technol.*.

[6] Schwarz S., Rupp M. (2016). Society in motion: challenges for LTE and beyond mobile communications. *IEEE Commun. Mag.*.

[7] Sheng J., Tang Z. W., Zhu Q. M., Wu C., Wang Y. M., Ai B. (2020). An improved interference alignment algorithm with user mobility prediction for high-speed railway wireless communication networks. *IEEE Access*.

[8] Yu N. F., Genevet P., Kats M. A. (2011). Light propagation with phase discontinuities: generalized laws of reflection and refraction. *Science*.

[9] Qian C., Chen H. (2021). A perspective on the next generation of invisibility cloaks – intelligent cloaks. *Appl. Phys. Lett.*.

[10] Ma J. G. (2021). From metamaterials to metadevices and applications. *IEEE Trans. Microw. Theory*.

[11] Cai T., Tang S., Zheng B. (2021). Ultrawideband chromatic aberration-free meta-mirrors. *Adv. Photon.*.

[12] Qian C., Zheng B., Shen Y. (2020). Deep-learning-enabled self-adaptive microwave cloak without human intervention. *Nat. Photon.*.

[13] Cai T., Zheng B., Lou J. (2022). Experimental realization of a superdispersion-enabled ultrabroadband terahertz cloak. *Adv. Mater.*.

[14] Luo L., Liu X., Duan S. (2023). Dual channel transformation of scalar and vector terahertz beams along the optical path based on dielectric metasurface. *Nanophotonics*.

[15] Dorrah A. H., Rubin N. A., Zaidi A., Tamagnone M., Capasso F. (2021). Metasurface optics for on-demand polarization transformations along the optical path. *Nat. Photon.*.

[16] Hu Q., Chen K., Zhao J., Dong S., Jiang T., Feng Y. (2022). On-demand dynamic polarization meta-transformer. *Laser Photon. Rev.*.

[17] Wang S., Deng Z. L., Wang Y. (2021). Arbitrary polarization conversion dichroism metasurfaces for all-in-one full poincaré sphere polarizers. *Light Sci. Appl.*.

[18] Hu Q., Chen K., Zhang N. (2022). Arbitrary and dynamic poincaré sphere polarization converter with a time-varying metasurface. *Adv. Opt. Mater.*.

[19] Qian C., Yang Y., Hua Y. (2022). Breaking the fundamental scattering limit with gain metasurfaces. *Nat. Commun.*.

[20] Wu Q., Zhang S., Zheng B., You C., Zhang R. (2021). Intelligent reflecting surface-aided wireless communications: a tutorial. *IEEE Trans. Commun.*.

[21] Fan Z., Qian C., Jia Y. (2022). Homeostatic neuro-metasurfaces for dynamic wireless channel management. *Sci. Adv.*.

[22] Shou Y., Feng Y., Zhang Y., Chen H., Qian H. (2022). Deep learning approach based optical edge detection using ENZ layers. *Prog. Electromagn. Res.*.

[23] Ma W., Chen W., Li D. (2023). Deep learning empowering design for selective solar absorber. *Nanophotonics*.

[24] Tan Qi., Qian C., Cai T., Zheng B., Chen H. (2022). Solving multivariable equations with tandem metamaterial kernels. *Prog. Electromagn. Res.*.

[25] Zhou E., Cheng Y., Chen F., Luo H., Li X. (2022). Low-profile high-gain wideband multi-resonance microstrip-fed slot antenna with anisotropic metasurface. *Prog. Electromagn. Res.*.

[26] Tsilipakos O., Tasolamprou A. C., Pitilakis A. (2020). Toward intelligent metasurfaces: the progress from globally tunable metasurfaces to software-defined metasurfaces with an embedded network of controllers. *Adv. Opt. Mater.*.

[27] Shaltout A. M., Lagoudakis K. G., van de Groep J. (2019). Spatiotemporal light control with frequency-gradient metasurfaces. *Science*.

[28] Zhang L., Chen X. Q., Liu S. (2018). Space-time-coding digital metasurfaces. *Nat. Commun.*.

[29] Chamanara N., Vahabzadeh Y., Caloz C. (2019). Simultaneous control of the spatial and temporal spectra of light with space-time varying metasurfaces. *IEEE Trans. Antenn. Propag.*.

[30] Dorrah A. H., Capasso F. (2019). Tunable structured light with flat optics. *Science*.

[31] Cao X., Chen Q., Tanaka T., Kozai M., Minami H. (2023). A 1-bit time-modulated reflectarray for reconfigurable-intelligent-surface applications. *IEEE Trans. Antenn. Propag.*.

[32] Li X., Yang H. Q., Shao R. W. (2022). Low cost and high performance 5-bit programmable phased array antenna at Ku-band. *Prog. Electromagn. Res.*.

[33] Liang J. C., Cheng Q., Gao Y. (2022). An Angle-Insensitive 3-bit reconfigurable intelligent surface. *IEEE Trans. Antenn. Propag.*.

[34] Zhang L., Cui T. J. (2019). Angle-insensitive 2-bit programmable coding metasurface with wide incident angles. *2019 IEEE Asia-Pacific Microwave Conference (APMC)*.

[35] Hu Q., Zhao J., Chen K. (2022). An intelligent programmable omni-metasurface. *Laser Photon. Rev.*.

[36] Fang X. Y., Li M., Han J. (2022). Accurate direction-of-arrival estimation method based on space-time modulated metasurface. *IEEE Trans. Antenn. Propag.*.

[37] Dai J. Y., Tang W., Wang M. (2022). Simultaneous in situ direction finding and field manipulation based on space-time-coding digital metasurface. *IEEE Trans. Antenn. Propag.*.

[38] Zhang N., Chen K., Hu Q. (2022). Spatiotemporal metasurface to control electromagnetic wave scattering. *Phys. Rev. Appl.*.

[39] Ramaccia D., Sounas D. L., Alu A., Toscano A., Bilotti F. (2017). Doppler cloak restores invisibility to objects in relativistic motion. *Phys. Rev. B*.

[40] Zhang X. G., Sun Y. L., Yu Q. (2021). Smart Doppler cloak operating in broad band and full polarizations. *Adv. Mater.*.

[41] Zhang J., Liu H., Wu Q. (2021). RIS-aided next-generation high-speed train communications: challenges, solutions, and future directions. *IEEE Wireless Commun.*.

[42] Jia Y. T., Qian C., Fan Z. (2022). In situ customized illusion enabled by global metasurface reconstruction. *Adv. Funct. Mater.*.

[43] Naseri P., Hum S. V. (2021). A generative machine learning-based approach for inverse design of multilayer metasurfaces. *IEEE Trans. Antenn. Propag.*.

[44] Chen J., Qian C., Zhang J., Jia Y., Chen H. (2023). Correlating metasurface spectra with a generation-elimination framework. *Nat. Commun.*.

[45] Li Z., Pestourie R., Park J., Huang Y., Johnson S. G., Capasso F. (2022). Inverse design enables large-scale high-performance meta-optics reshaping virtual reality. *Nat. Commun.*.

[46] Zhang J., Qian C., Fan Z. (2022). Heterogeneous transfer-learning-enabled diverse metasurface design. *Adv. Opt. Mater.*.

[47] Qu K., Chen K., Hu Q., Zhao J., Jiang T., Feng Y. (2023). Deep-learning-assisted inverse design of dual-spin/frequency metasurface for quad-channel off-axis vortices multiplexing. *Adv. Photon. Nexus*.

[48] Lecun Y., Bengio Y., Hinton G. (2015). Deep learning. *Nature*.

[49] Ma W., Cheng F., Liu Y. M. (2018). Deep-learning-enabled on-demand design of chiral metamaterials. *ACS Nano*.

[50] Liu W. B., Wang Z. D., Liu X. H., Zengb N. Y., Liu Y. R., Alsaadi F. E. (2017). A survey of deep neural network architectures and their applications. *Neurocomputing*.

[51] Jia Y. T., Qian C., Fan Z. X., Cai T., Li E. P., Chen H. S. (2023). A knowledge-inherited learning for intelligent metasurface design and assembly. *Light Sci. Appl.*.

[52] Succetti F., Rosato A., Di Luzio F., Ceschini A., Panella M. (2022). A fast deep learning technique for Wi-Fi-based human activity recognition. *Prog. Electromagn. Res.*.

[53] Liu D. J., Tan Y. X., Khoram E., Yu Z. F. (2018). Training deep neural networks for the inverse design of nanophotonic structures. *ACS Photonics*.

[54] Zhu X. Y., Qian C., Jia Y. (2023). Realization of index modulation with intelligent spatiotemporal metasurfaces. *Adv. Intell. Syst.*.

[55] Zhen Z., Qian C., Jia Y. (2021). Realizing transmitted metasurface cloak by a tandem neural network. *Photon. Res.*.

[56] Huang M., Zheng B., Cai T. (2022). Machine–learning-enabled metasurface for direction of arrival estimation. *Nanophotonics*.

[57] Wang Z., Qian C., Cai T. (2021). Demonstration of spider-eyes-like intelligent antennas for dynamically perceiving incoming waves. *Adv. Intell. Syst.*.

[58] Lu H., Zhao J., Zheng B. (2023). Eye accommodation-inspired neuro-metasurface focusing. *Nat. Commun.*.

