# Supplementary material for: On-demand Doppler-offset beamforming with intelligent spatiotemporal metasurfaces
Source: Nanophotonics. 2023 Nov 17;13(8):1351–60. doi: 10.1515/nanoph-2023-0569 (PMC11636439; doi:10.1515/nanoph-2023-0569)
Supplement: Supplementary file 1 — Supplementary Material Details [file j_nanoph-2023-0569_suppl_001.docx]

**On-demand Doppler-offset beamforming with intelligent spatiotemporal metasurfaces**

Xiaoyue Zhu^1,2,3^, Chao Qian^1,2,3,*^, Jie Zhang^1,2,3^,yuetian Jia^1,2,3^, Yaxiong Xu^1,2,3^, Mingmin Zhao^4^, Minjian Zhao^4^, Fengzhong Qu^5^, and Hongsheng Chen^1,2,3,*^

*^1^ ZJU-UIUC Institute, Interdisciplinary Center for Quantum Information, State Key Laboratory of Extreme Photonics and Instrumentation, Zhejiang University, Hangzhou 310027, China.*

*^2^ZJU-Hangzhou Global Science and Technology Innovation Center, Key Lab. of Advanced Micro/Nano Electronic Devices & Smart Systems of Zhejiang, Zhejiang University, Hangzhou 310027, China.*

*^3^ Jinhua Institute of Zhejiang University, Zhejiang University, Jinhua 321099, China.*

*^4^ Department of Information Science and Electronic Engineering, Zhejiang University Hangzhou 310027, China.*

*^5^ Ocean College Zhejiang University, Zhoushan 316021, China.*

*^*^Corresponding author:* [*chaoq@intl.zju.edu.cn*](mailto:chaoq@intl.zju.edu.cn) *(C. Qian);* [*hansomchen@zju.edu.cn*](mailto:hansomchen@zju.edu.cn) *(H. Chen)*

**Supplementary Note 1：Metasurface design and its reflection spectra**

For further practical verifications, we meticulously design a basic unit cell employing square element [28], [30]. Figure S1a provides an incisive depiction of the sophisticated configuration with the dimensions subx = suby = 42.86 mm, H = 2 mm, px = py = 25.8 mm, w = 1 mm, and r = 1 mm. The judicious application of a control voltage (either high or low) to the bias line regulates the state of the integrated PIN diode, switching it between the ON and OFF states and eliciting a corresponding modulation in the reflection characteristics [34], [35]. To assess the reconfigurable metasurfaces, we undertook experimental measurements to catalog the reflection amplitude and phase in the ON/OFF state, as depicted in Figs. S1b and S1c. Notably, our empirical data accentuates that the most salient phase variations are concentrated around 3.5 GHz. As a result, this frequency is designated as the epicenter of our investigation. At this frequency, the reflection phase and amplitude for the ON and OFF states were measured to be approximately -41.89° and 64.97°, and 0.87 and 0.90 (average), respectively. By the way, the cell is a little sensitive to incident angles. This can be easily solved by measuring spectra under different incident angles in advance. Besides, more angle insensitive cell structures[S1], [S2] can be adopted, as the spatiotemporal modulation has no restrictions on the structures of tunable metasurfaces.


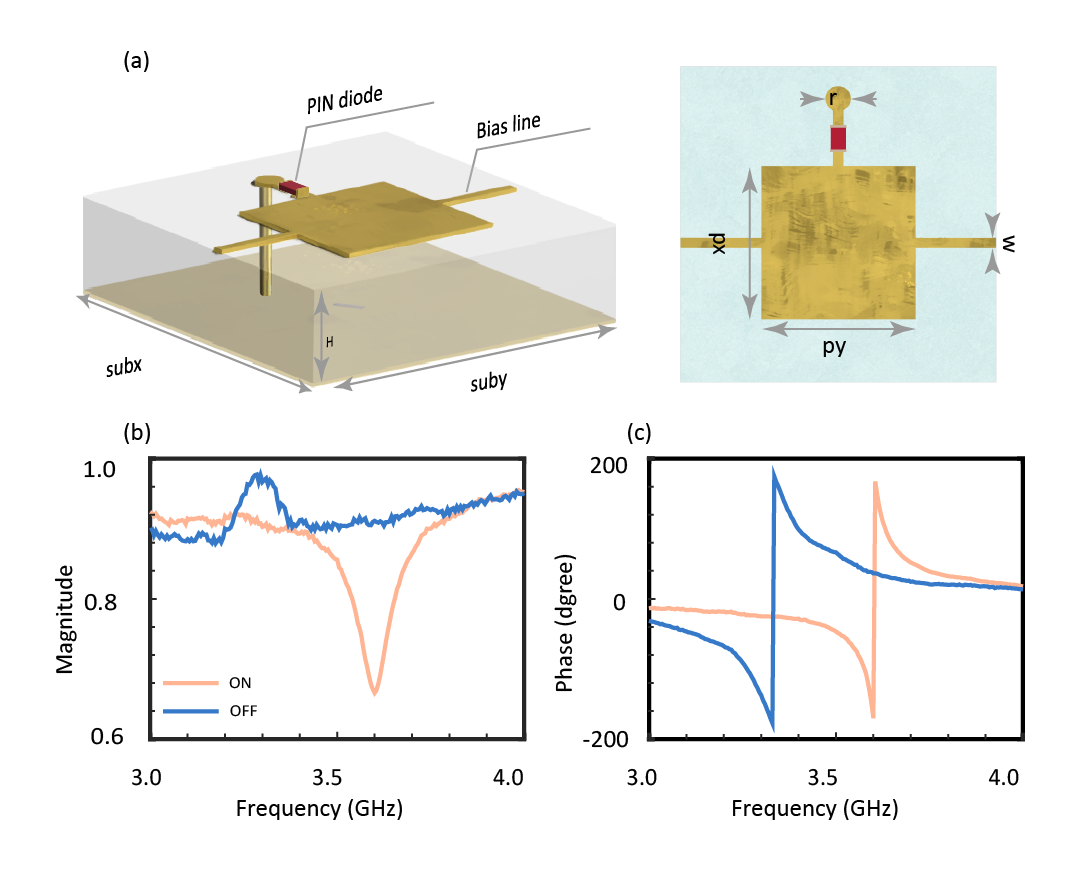


**Fig. S1**| Structural design of tuanble metasurfaces. (a), Overview of the unit cell structure and corresponding geometric parameters. (b), Measured reflection magnitude. (c), Measured reflection phases.

**Supplementary Note 2：Used targets**

Following the initial tests, we proceeded to conduct another experiment to ascertain the ability of the spatiotemporal metasurfaces to manipulate beam directionality under vertical incidence. In this experiment, the target beamforms were configured to peak in the directions of $\theta=\pm45^{\circ}$. And the target is constructed via taking absolute value and shifting the center of rolling cosine functions whose rolling coefficients are 0.5. The configuration of the targets is depicted in Figs. S2a and S2b, where the pink lines represent the fields predicted by the network, while the blue lines signify the fields calculated numerically. For the sake of consistency and convenience, we opted to use the +1st harmonic as a representative example for demonstration purposes. The receiver was strategically placed at these two directional positions to capture the reflected harmonics. The spectrum of the signals received was then plotted in the main text. To augment the evaluation of prediction accuracy, the Pearson Correlation Coefficients (PCC) were also computed. The ensuing data revealed an average PCC surpassing 90%, thereby affirming the network’s predictions to be of high precision and dependability.


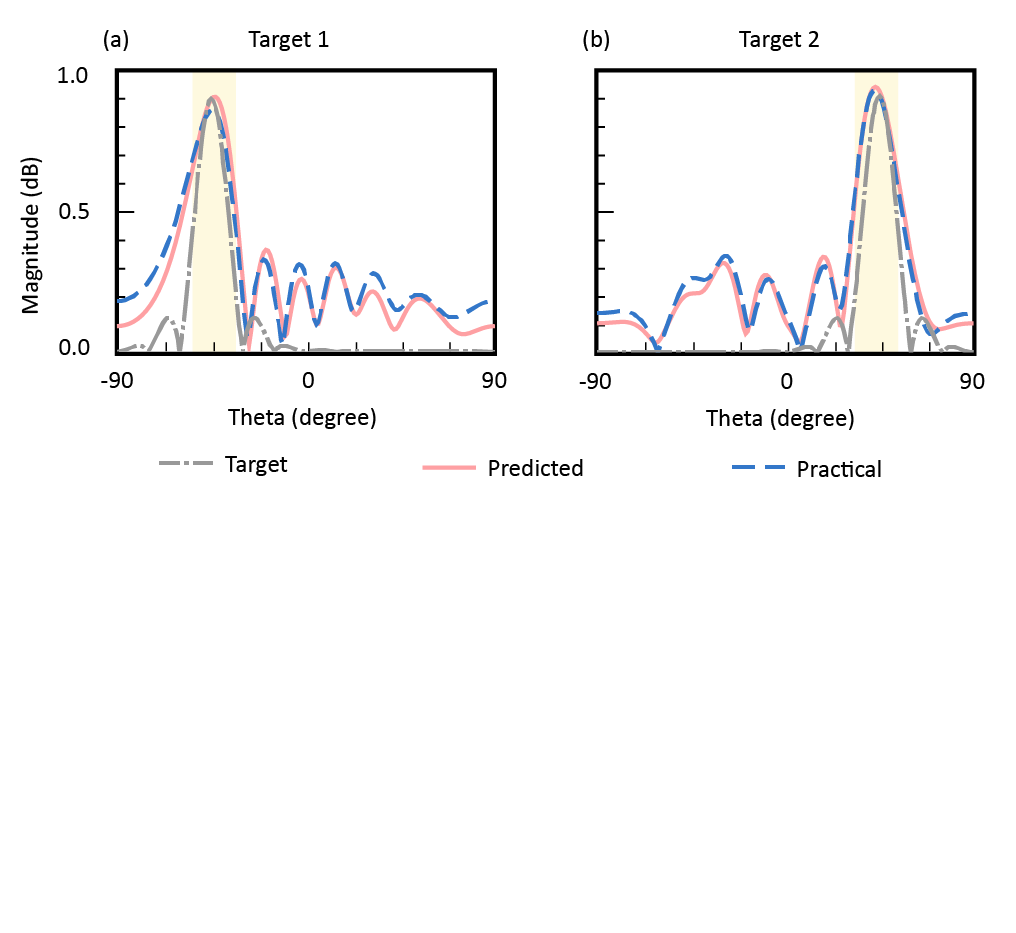


**Fig. S2 |** Experimental results in vertical incident scenes. The left figure is received at $\theta=+45^{\circ}$ and the right one is received at $\theta=-45^{\circ}$.

**Supplementary Note 3：System descriptions**

The "on-demand" and "intelligent" process are fulfilled by the tandem neural network, which builds up a fast channel between the time-varying series of metasurfaces and the target far-fields. The whole network is constructed by connecting an inverse design network with a forward design network. In the working process, the whole network is striving to make the predicted far-field similar to the desired input far-field. In this manner, the designed time-varying series can be extruded from the mid layer of the whole network. The target far-field can be automatically generated in real time based on application demands, such as beam directions and widths. The frequency shift is decided by time-varying series length and clock frequency which can be alter by changing clock frequencies. The environmental information like incoming wave directions, angles and frequencies can be detected via previously designed detectors [S3].

**References**

[S1] J. C. Liang *et al*., "An Angle-Insensitive 3-Bit Reconfigurable Intelligent Surface," in *IEEE Trans. Antenn. Propag.*, vol. 70, no. 10, pp. 8798-8808, 2022.

[S2] L. Zhang and T. J. Cui, "Angle-Insensitive 2-Bit Programmable Coding Metasurface with Wide Incident Angles," *2019 IEEE Asia-Pacific Microwave Conference (APMC)*, Singapore, 2019, pp. 932-934

[S3] Z. Wang et al., "Demonstration of spider-eyes-like intelligent antennas for dynamically perceiving incoming waves," *Adv. Intell. Syst.,* vol. 3, no. 9, pp. 2100066, 2021.
